# Validation of aortic valve pressure gradient quantification using semi-automated 4D flow CMR pipeline

**DOI:** 10.1186/s13104-022-06033-z

**Published:** 2022-04-29

**Authors:** Ciaran Grafton-Clarke, Paul Njoku, Jean-Paul Aben, Leon Ledoux, Liang Zhong, Jos Westenberg, Andrew Swift, Gareth Archer, James Wild, Rod Hose, Marcus Flather, Vassilios S. Vassiliou, Pankaj Garg

**Affiliations:** 1 Norwich Medical School, University of East Anglia, Norwich, S10 2RX UK; 2 Pie Medical Imaging, Maastricht, The Netherlands; 3 National Heart Centre, Singapore, Singapore; 4 Leids Universitair Medisch Centrum, Leiden, The Netherlands; 5 University of Sheffield, Sheffield, UK

**Keywords:** Aortic stenosis, Cardiac MRI, Validation

## Abstract

**Objective:**

Doppler echocardiographic aortic valve peak velocity and peak pressure gradient assessment across the aortic valve (AV) is the mainstay for diagnosing aortic stenosis. Four-dimensional flow cardiovascular magnetic resonance (4D flow CMR) is emerging as a valuable diagnostic tool for estimating the peak pressure drop across the aortic valve, but assessment remains cumbersome.

We aimed to validate a novel semi-automated pipeline 4D flow CMR method of assessing peak aortic value pressure gradient (AVPG) using the commercially available software solution, CAAS MR Solutions, against invasive angiographic methods.

**Results:**

We enrolled 11 patients with severe AS on echocardiography from the EurValve programme. All patients had pre-intervention doppler echocardiography, invasive cardiac catheterisation with peak pressure drop assessment across the AV and 4D flow CMR.

The peak AVPG was 51.9 ± 35.2 mmHg using the invasive pressure drop method and 52.2 ± 29.2 mmHg for the 4D flow CMR method (semi-automated pipeline), with good correlation between the two methods (r = 0.70, p = 0.017).

Assessment of AVPG by 4D flow CMR using the novel semi-automated pipeline method shows excellent agreement to invasive assessment when compared to doppler-based methods and advocate for its use as complementary to echocardiography.

**Supplementary Information:**

The online version contains supplementary material available at 10.1186/s13104-022-06033-z.

## Introduction

Aortic stenosis (AS) is the most common valvular heart disease [1], affecting nearly 10% of people over the age of 80 years [2]. With its prevalence increasing with age, 2 year survival rates can be less than 50% in symptomatic subjects with reduced cardiac function, unless the valve is replaced [3]. With no medical therapy proven to attenuate progression or improve survival [4], temporising balloon valvuloplasty may be required as a bridge to a definitive valvular replacement, which is performed either surgically (SAVR) or using a transcatheter approach (TAVR). Balancing the risk between operative mortality and post-replacement complications against the likelihood of disease progression and cardiovascular death is essential in determining the appropriateness and timing of intervention. Several imaging modalities can be used to evaluate valve anatomy, haemodynamics, and the morphological sequelae of disease, which alongside objective measures of functional limitation, can be used to grade severity.

Transthoracic echocardiography (TTE) is the first-line investigation to assess the severity of AS and its impact on haemodynamics and LV function [5].

Four-dimensional (4D) flow cardiovascular magnetic resonance (CMR) imaging is emerging as a valuable non-invasive imaging technique that circumvents many of the echocardiographic limitations such as Doppler misalignment and geometric assumptions [6]. Validation studies on manual 4D flow CMR assessment demonstrated comparable estimates between 4D flow CMR and invasive assessment methods but highlighted the requirement for significant acquisition and post-processing competence [7]. Intending to progress the evolution of advanced imaging techniques into streamlined tools for clinical practice, we aimed to validate the 4D flow CMR (semi-automated pipeline) method of assessing peak aortic valve pressure gradient (AVPG) against the reference invasive pressure drop method and two other methods of peak AVPG quantification (TTE and 4D flow CMR with the manual assessment of AVPG). We also performed reproducibility analyses for the novel semi-automated pipeline 4D flow CMR method of peak AVPG assessment.

## Main text

### Materials and methods

#### Study population

11 subjects from the EurValve programme with suspected severe aortic stenosis on echocardiography were included [7]. All subjects had pre-TAVI/SAVR peak AVPG assessment with TTE, invasive cardiac catheterisation, and 4D flow CMR.

#### Ethics

Ethical approval was provided by the National Research Ethics Service, United Kingdom (17/LO/0283). Informed written consent was obtained from all subjects.

#### Non-CMR assessment of peak AVPG

Transthoracic echocardiography was performed according to the British Society of Echocardiography guidelines [8]. Severity grading of aortic stenosis was performed as per the European Society of Cardiology (ESC) consensus guidelines [5].

Invasive pressure gradients were obtained during cardiac catheterisation in all patients as part of routine care before TAVI/SAVR. Full details of the procedure to invasively assess peak AVPG are presented in Additional file 1: (item 1) and previously published reports [7]. Manual 4D flow CMR assessment was performed in MASS (version 2019 EXP, LUMC Netherlands). The semi-automated pipeline 4D flow CMR assessment was performed in CAAS (prototype version 5.2.1, Pie Medical Imaging, the Netherlands).

#### CMR protocol

CMR was performed on a 3.0 Tesla Phillips Healthcare Ingenia system equipped with a 28-channel coil and Phillips dStream digital broadband MR architecture technology.

The CMR protocol included baseline surveys, cines (vertical long-axis, horizontal long-axis, short-axis contiguous left-ventricle volume stack, 3-chamber, and aortic root). Cine images were acquired during end-expiratory breath-holds with a balanced steady-state free precession, single-slice breath-hold sequence. Procedures relating to 4D flow CMR pre-processing were delivered in accordance with established standards of practice [9]. Technical parameters for the CMR protocol are presented in Additional file 1: (items 2 and 3).

#### 4D flow CMR assessment of AVPG

Post-processing and analysis were computed using CAAS MR Solutions (prototype version 5.2.1, Pie Medical Imaging, the Netherlands). Two assessors with at least two years of CMR experience independently calculated peak AVPG using the novel semi-automated pipeline method (CGC, PG). CGC repeated the analysis after four weeks, blinded to the results of the first analysis for intraobserver reproducibility analysis. When calculating peak AVPG, the assessors was blinded from the results of TTE and invasive assessments.

Two methods involving 4D flow CMR were used to determine peak AVPG. The manual method, as described in Archer et al. [7], and a novel semi-automated pipeline method. For both methods, aliasing correction was performed automatically in all three-phase directions. Any spatial misalignment with cine superimposition was manually corrected throughout the cardiac cycle before quantification.

The manual method involved plotting several multi-planar slices above the valvular plane to assess the quality of flow curves in the region of the vena contracta. The reformatted plane with the highest velocity and no artefact was then used to determine the peak pressure drop, calculated using the simplified Bernoulli Eq. (4V_max_^2^). This method is rigorously described within a previously published report [7].

**Fig. 1:**
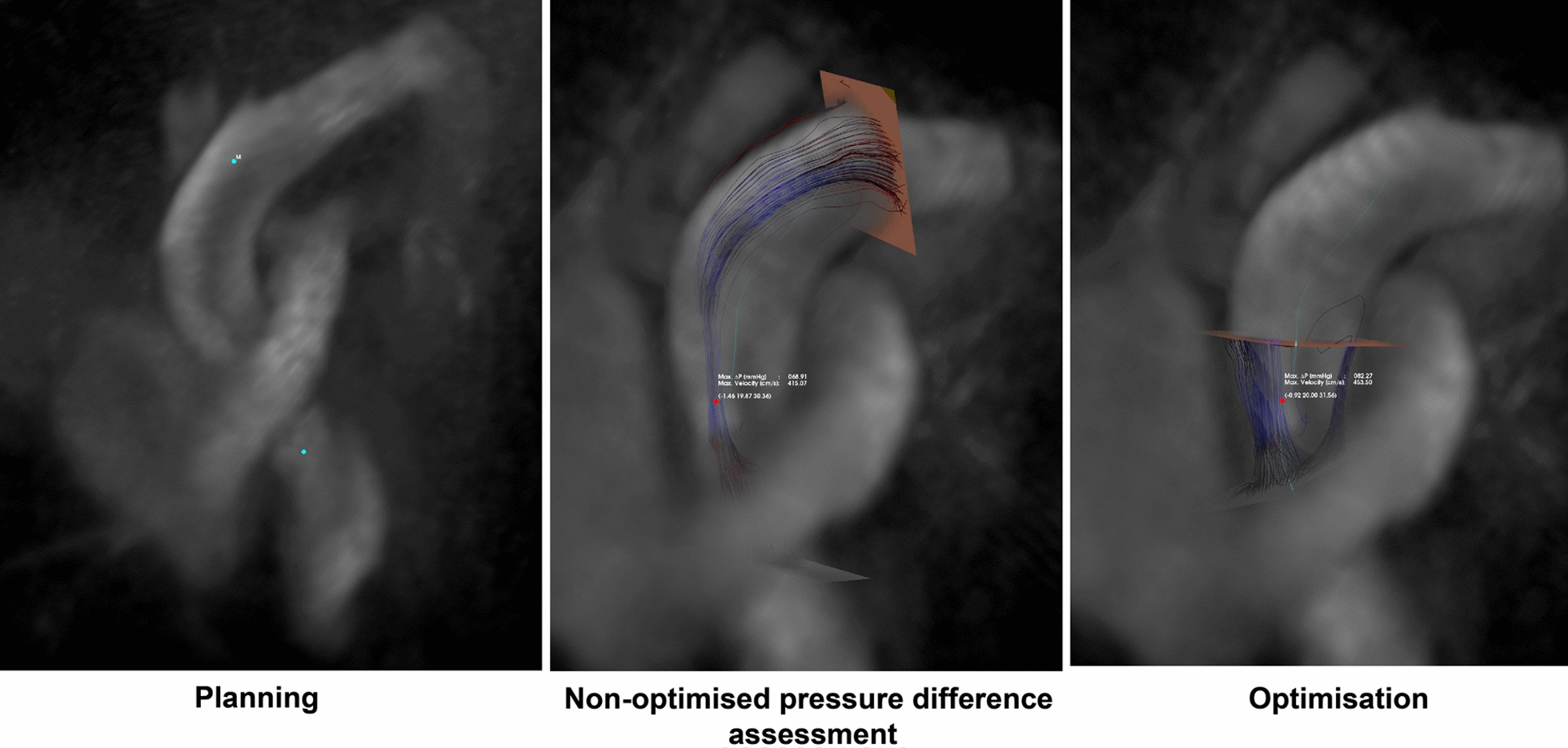
Semi-automated pipeline 4D flow CMR assessment of peak AVPG post-processing steps. Planning stage—after loading the 4D flow in CAAS MR Solution, two points are defined in the weighted reconstructed three-dimensional image, one just below the aortic value and the other within the ascending aorta. Non-optimised pressure difference assessment—this is the starting point to explore the location of the true peak velocity during systole. Optimisation—the two planes are optimised ensuring the aortic valve panel is angulated appropriately

The novel automated pipeline 4D flow CMR evaluation of AVPG involved three steps which are detailed in Fig. 1.

#### Statistical analysis

All continuous parameters are reported as mean ± standard deviation (SD). Correlation between TTE, 4D flow CMR (manual method), 4D flow CMR (semi-automated pipeline method) and the reference invasive pressure drop assessment were calculated using Pearson’s correlation coefficient. Agreement between the four methods was calculated using Bland–Altman statistics, where the mean difference between the two methods was reported as the relative risk of bias (measured in mmHg). The Shapiro–Wilk test assessed the normality of data. Since the data was normally distributed, parametric two-tailed paired samples t-test were used between peak AVPG assessment using invasive measures compared with TTE and the two 4D flow CMR methods. Interobserver and intraobserver variability were assessed using the interclass correlation coefficient (ICC). For all analyses, p < 0.05 was deemed to be statistically significant.

## Results

11 subjects underwent peak AVPG assessment using TTE, cardiac catheterisation, and 4D flow CMR. The mean age of subjects was 80 ± 6 years. All subjects were male (n = 11). The mean NYHA class of subjects was 2 (range 1–3). Detailed clinical and imaging characteristics are provided in Additional file 1: (item 4). All CMR studies were of good imaging quality and there were no notable technical difficulties in 4D flow CMR analyses.

The mean peak AVPG was 51.9 ± 35.2 mmHg using the invasive pressure drop method. The mean peak AVPG was 66.7 ± 34.8, 53.7 ± 23.6 and 52.2 ± 29.2 mmHg for TTE, 4D flow CMR (manual method) and 4D flow CMR (semi-automated pipeline method), respectively (Additional file 1: item 5).

### Validation of AVPG assessment using 4D flow CMR (novel automated pipeline)

The mean peak pressure gradient derived from the semi-automated pipeline 4D flow CMR method was comparable to the invasive pressure gradient method (52.2 ± 29.2 versus 51.9 ± 35.2 mmHg) with minimal bias between the two methods (bias = 0.3 mmHg, p = 0.974). 4D flow CMR (semi-automated pipeline) method of peak AVPG showed good correlation to the invasive pressure drop method (r = 0.70, p = 0.017) (Table 1).

**Table 1:** Correlation and agreement analysis in peak AVPG assessment between invasive cardiac catheterisation and three other methods

	Transthoracic echocardiography		4D flow CMR (manual method)		4D flow CMR (semi-automated pipeline method)	
	r	Bias (mmHg)	r	Bias (mmHg)	r	Bias (mmHg)
Invasive cardiac catheterisation	0.95 (p < 0.001)	−14.8 (p = 0.001)	0.63 (p = 0.04)	−1.8 (p = 0.836)	0.70 (p = 0.02)	−0.3 (p = 0.974)

Correlation analysis using the Pearson correlation coefficient (denoted r) and agreement analysis using Bland–Altman statistics (denoted Bias). For agreement analysis, bias refers to the mean difference between the two methods of peak AVPG assessment (measured in mmHg) and is deemed statistically significant if the corresponding p-valve (denoted p) is < 0.05 (i.e., high risk of systematic bias). For negative bias values, this indicates that the non-invasive method (either TTE or 4D flow CMR) for peak AVPG assessment is systematically lower than the values derived from the invasive method

### Correlation analyses

**Fig. 2:**
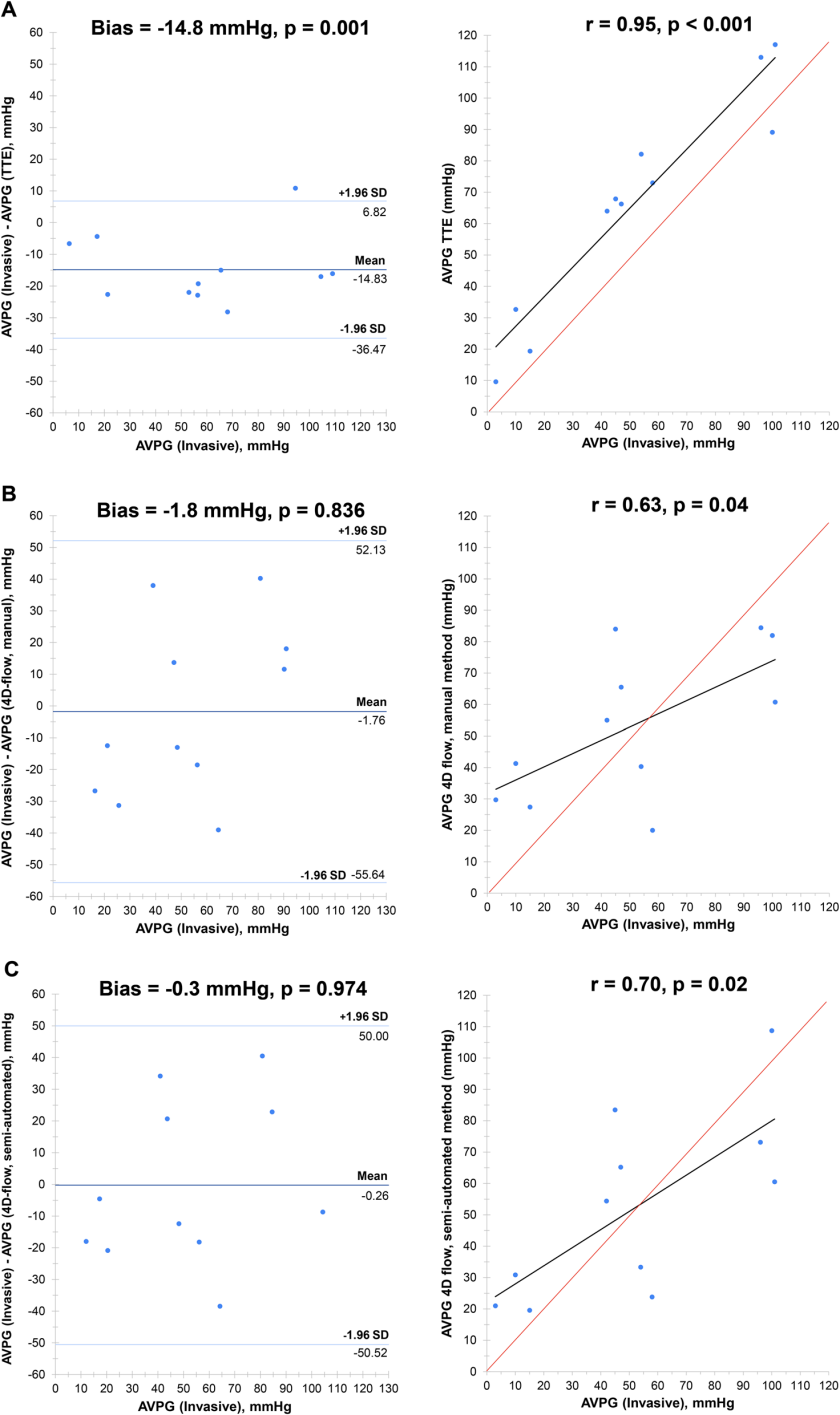
Agreement and correlation analyses between invasively derived peak aortic valve pressure gradient assessment and three other methods. Agreement and correlation analyses between invasively derived peak aortic valve pressure gradients (AVPG) and** A**) transthoracic echocardiography (TTE);** B**) 4D flow CMR (manual method);** C**) 4D flow CMR (semi-automated pipeline method). Left panel relates to agreement analyses (Bland–Altman statistics), where bias refers to the mean difference between the two methods of peak AVPG assessment (measured in mmHg) and is deemed statistically significant if the corresponding p-valve (denoted p) is < 0.05 (i.e., high risk of systematic bias). For negative bias values, this indicates that the non-invasive method (either TTE or 4D flow CMR) for peak AVPG assessment is systematically lower than the values derived from the invasive method. The right panel relates to correlation analyses between the reference invasive method of peak AVPG assessment and TTE, 4D flow CMR (manual method) and 4D flow CMR (semi-automated pipeline method) derived values. The Pearson correlation coefficient is denoted r, with accompanying p-values (denoted p). The line of best fit (black) and r = 1 (red) is presented

Compared with invasive assessment, the most strongly correlated method of peak pressure gradient was TTE (r = 0.95, p < 0.001) followed by 4D flow CMR (semi-automated pipeline method; r = 0.70, p = 0.02) and then 4D flow CMR (manual method; r = 0.63, p = 0.04) (Fig. 2). The two methods of 4D flow CMR derived peak pressure gradient were strongly correlated to each other (r = 0.94, p < 0.001), but showed modest correlation to TTE (manual method, r = 0.61, p = 0.04; semi-automated pipeline method, r = 0.62, p = 0.04).

### Agreement analyses

Compared with invasive assessment, the method demonstrating the lowest bias was 4D flow CMR semi-automated pipeline method (bias = −0 .3 mmHg, p = 0.974) followed by 4D flow CMR manual method (bias = 1.8 mmHg, p = 0.836) (Fig. 2). TTE demonstrated significant bias when compared to invasively derived peak pressure gradients (bias = 14.8 mmHg, p = 0.001).

### Interobserver and intraobserver reproducibility

Interobserver reproducibility analysis between two independent assessors demonstrated excellent agreement in peak pressure gradient assessment using the semi-automated pipeline 4D flow CMR method (ICC 0.98, 95% CI 0.92–0.99).

Intraobserver reproducibility analysis between two blinded readings from one assessor demonstrated excellent agreement in peak pressure gradient assessment using the semi- automated pipeline 4D flow CMR method (ICC 0.99, 95% CI 0.99–1.00).

## Discussion

This study provides the first validation of a novel semi-automated pipeline method of aortic valve peak pressure gradient assessment by 4D flow CMR against transthoracic echocardiography and the reference standard, cardiac catheterisation, for subjects with severe aortic stenosis. We have identified that automated pipeline aortic valve peak pressure gradient assessment by 4D flow CMR demonstrates superior agreement to invasively measured pressure gradient when compared to echocardiography and 4D flow CMR (manual method). Consistent with other studies [7, 10], we also identified a systematic bias between Doppler and invasive measures for the assessment of peak pressure drop across the aortic valve, and therefore recommend the use of 4D flow CMR to corroborate and sense-check the metrics from echocardiography in challenging or discrepant cases. It is important to reinforce that echocardiography should remain the key technique through which the diagnosis of valvular heart diseases is screened, and the progression of disease is monitored. Echocardiography, in the assessment of thin and mobile valve leaflets, offers excellent spatial and temporal resolution, which considered alongside its low cost and accessibility, makes it an incredibly valuable diagnostic tool in patients with suspected aortic disease. 4D flow CMR assessment has clear benefits. For instance, visualisation of blood flow (i.e., streamlines / pathlines) permits the modelling of complex 3D flow jet patterns and haemodynamic changes associated with aortic stenosis [6]. An additional benefit of 4D flow CMR is the ability to retrospectively and flexibly visualise and quantify blood flow without being restricted to 2D plane and view angles. Since it is common for systolic flow jets in AS to be eccentric, 4D flow CMR offers a significant benefit over TTE in that it is not hindered by flow eccentricity [11]. 4D-flow CMR offers multidirectional velocity-encoding which allows for quantification of velocities regardless of the spatial orientation of the flow jet.

A recent cohort study comparing 2D and 4D flow CMR with TTE in patients with severe aortic stenosis showed that 4D flow CMR significantly underestimates systolic peak flow velocity [12]. Our study provides a different perspective, as we included a comparison with the gold-standard reference, invasive assessment. Our study suggests it is rather that TTE overestimates systolic peak flow volume, rather than 4D flow CMR underestimating it, as the latter demonstrates excellent concordance to invasive assessment with minimal bias. It is recognised that peak aortic jet velocity assessment using echocardiography is user dependent and carries several pitfalls that may result in under- or overestimated of stenosis severity [13]. For instance, misalignment of the Doppler beam with the AS jet risks substantial underestimation of aortic velocity. A further limitation of echocardiography is that aortic jet velocity is highly flow dependent. This has the potential to lead to profound overestimation of AS severity in high-flow states (e.g., concomitant aortic regurgitation, severe anaemia, or thyrotoxicosis). Other common limitations to echocardiography include poor acoustic quality,

In addition to the comparable agreement and precision of the semi-automated pipeline 4D flow CMR method to the manual 4D flow CMR method, there was a notable difference in the processing time to obtain the peak AVPG value in the former, taking on average 4 min per subject to quantify, in comparison to 20 min using the equivalent manual 4D flow CMR method. This operational outcome, alongside the reduced technical and post-processing competence required of the operator in using the semi-automated pipeline method, is notable, given the need to streamline and simplify the complex acquisition and post-processing procedures for routine clinical practice.

## Limitations

We included only a small number of subjects included within the analyses. It is important for larger studies to be performed to corroborate and evolve our findings. There is a need for same-day dual-modality echo-MRI studies to allow for greater confidence in the results of agreement analyses, in addition to determining the performance of techniques, either in isolation or in tandem, within the clinical setting. Caution also needs to be made to the fact the manual 4D flow CMR assessment of peak AVPG was performed in MASS (version 2019 EXP, LUMC, The Netherlands), whereas the semi-automated pipeline 4D flow CMR method was used within CAAS. The former is not licensed for commercial use, whereas CAAS is. Furthermore, there have been no studies evaluating the concordance between manual peak AVPG assessment between CAAS and MASS. MASS does not currently offer a semi-automated pipeline method for peak AVPG assessment.

## Supplementary Information

**Additional file 1**: Item 1: Invasive pressure gradient assessment. Item 2: CMR protocol technical parameters. Item 3: 4D flow CMR acquisition procedures. Item 4: Baseline characteristics. Item 5: Peak aortic value pressure gradient across the four methods of assessment.

## Data Availability

The datasets generated and analysed during the current study are available in the Harvard repository, https://doi.org/10.7910/DVN/OLOH39.

## Abbreviations

4D: Four dimensional
AS: Aortic stenosis
AVPG: Aortic valve pressure gradient
CMR: Cardiovascular magnetic resonance
ESC: European Society of Cardiology
ICC: Interclass correlation coefficient
NYHA: New York heart association
SAVR: Surgical aortic valve replacement
SD: Standard deviation
TAVR: Trans-aortic valve replacement
TTE: Transthoracic echocardiography
