# Supplementary material for: Validation of aortic valve pressure gradient quantification using semi-automated 4D flow CMR pipeline
Source: BMC Res Notes. 2022 Apr 29;15:151. doi: 10.1186/s13104-022-06033-z (PMC9052497; doi:10.1186/s13104-022-06033-z)
Supplement: Supplementary file 1 — Additional file 1: Item 1: Invasive pressure gradient assessment. Item 2: CMR protocol technical parameters. Item 3: 4D flow CMR acquisition procedures. Item 4: Baseline characteristics. Item 5: Peak aortic value pressure gradient across the four methods of assessment. [file 13104_2022_6033_MOESM1_ESM.docx]

**Item 1: Invasive pressure gradient assessment**

Cardiac catheterisation was performed via the femoral artery using standard techniques. Seven-French pigtail catheters were placed in the ascending aorta and the LV cavity and pressures recorded simultaneously. Analysis was performed by the Xper CardioFlex system (Phillips Healthcare, The Netherlands). Peak-to-peak pressure gradient was determined in millimetres of mercury (mmHg). Note, subjects were not eligible for inclusion if invasive pressure gradient assessment was not recordable.

**Item 2: CMR protocol technical parameters**

Cine images had a spatial resolution of 2.5 × 2.5 mm^2^, interpolated to 1.56 × 1.56 mm^2^, and a slice thickness of 10 mm with contiguous slices for the short axis stack. Other imaging parameters were 30 phases, echo time (TE) = 1.5 ms, repetition time (TR) = 3.05 ms, flip angle = 45°, the field of view (FOV) was 400 mm, and SENSE factor 2–3.

**Item 3: 4D flow CMR acquisition procedures**

The initial VENC settings were estimated from TTE peak velocity and tested using a through-plane two-dimensional phase-contrast acquisition. Further increments were added until aliasing disappeared across the aortic valve. FOV was planned to cover the entire heart, aortic valve, and descending aorta. The 4D flow sequence used echo-planar imaging (EPI) acceleration factory of 5 with no respiratory gating. Other scan parameters were acquired voxel size = 3 x 3 x 3 mm, reconstructed voxel size = 1.5 x 1.5 x 1.5 mm, echo time (TE) = 3.5 ms, repetition time (TR) = 10 ms, flip angle 10°, FOV 340 x 340 mm, and 30 phases for each cardiac cycle.

Data pre-processing was performed on the scanner to correct for phase offset errors such as eddy currents, Maxwell effects, and encoding errors related to gradient field distortions to avoid impairment of the measurements and inaccuracies in flow quantification.

**Item 4: Baseline characteristics**

| **Demographic** | |
| --- | --- |
| Age (years) | 80.0 ± 6.8 |
| Gender, male (%) | 100.0 |
| Sinus rhythm, n (%) | 6 (54.5) |
| **Comorbidities** | |
| Type II diabetes mellitus, n (%) | 3 (27.3) |
| Hypertension, n (%) | 9 (81.8) |
| Previous myocardial infarction, n (%) | 0 (0.0) |
| Smoker, n (%) | 3 (27.3) |
| NYHA classification | 2 |
| **Functional data** | |
| LVEDV (ml) | 122.3 ± 27.5 |
| LVESV (ml) | 51.4 ± 18.3 |
| LVSV (ml) | 70.9 ± 11.0 |
| LVMass (g) | 129.3 ± 34.9 |
| LVEF (%) | 59.1 ± 6.9 |
| **Subsequent treatment** | |
| TAVI, n (%) | 10 (90.9) |
| SAVR, n (%) | 1 (9.9) |

Values are mean ± SD.

LVEDV – left ventricle end-diastolic volume; LVESV – left ventricle end-systolic volume; LVEF – left ventricle ejection fraction; LVSV – left ventricle stroke volume; NYHA – New York Heart Association; SAVR – surgical aortic valve replacement; TAVI – transcatheter aortic valve insertion

**Item 5: Peak aortic value pressure gradient across the four methods of assessment**

|  | TTE | Cardiac catheterisation | 4D flow CMR (manual) | 4D flow CMR (semi-automated) |
| --- | --- | --- | --- | --- |
| Peak aortic value pressure gradient, mean (mmHg) ± SD | 66.7 ± 34.8 | 51.9 ± 35.2 | 53.7 ± 23.6 | 52.2 ± 29.2 |
